# PSTPIP1-LYP phosphatase interaction: structural basis and implications for autoinflammatory disorders

**DOI:** 10.1007/s00018-022-04173-w

**Published:** 2022-02-12

**Authors:** José A. Manso, Tamara Marcos, Virginia Ruiz-Martín, Javier Casas, Pablo Alcón, Mariano Sánchez Crespo, Yolanda Bayón, José M. de Pereda, Andrés Alonso

**Affiliations:** 1 Unidad de Excelencia Instituto de Biología y Genética Molecular (IBGM), CSIC-Universidad de Valladolid, c/ Sanz y Forés 3, 47003 Valladolid, Spain; 2 Instituto de Biología Molecular y Celular del Cáncer (IBMCC), CSIC-Universidad de Salamanca, Campus Unamuno, 37007 Salamanca, Spain

**Keywords:** Immunology, Auto-inflammation, LYP, PSTPIP1

## Abstract

Mutations in the adaptor protein PSTPIP1 cause a spectrum of autoinflammatory diseases, including PAPA and PAMI; however, the mechanism underlying these diseases remains unknown. Most of these mutations lie in PSTPIP1 F-BAR domain, which binds to LYP, a protein tyrosine phosphatase associated with arthritis and lupus. To shed light on the mechanism by which these mutations generate autoinflammatory disorders, we solved the structure of the F-BAR domain of PSTPIP1 alone and bound to the C-terminal homology segment of LYP, revealing a novel mechanism of recognition of Pro-rich motifs by proteins in which a single LYP molecule binds to the PSTPIP1 F-BAR dimer. The residues R228, D246, E250, and E257 of PSTPIP1 that are mutated in immunological diseases directly interact with LYP. These findings link the disruption of the PSTPIP1/LYP interaction to these diseases, and support a critical role for LYP phosphatase in their pathogenesis.

**Supplementary Information:**

The online version contains supplementary material available at 10.1007/s00018-022-04173-w.

## Introduction

Pyogenic arthritis, pyoderma gangrenosum and acne (PAPA) is a rare autoinflammatory disease caused by mutations in the gene that codes for the proline–serine–threonine phosphatase interacting protein 1 (PSTPIP1) [1]. PAPA is characterized by the presence of sterile arthritis with a rich neutrophilic infiltrate in the joints, ulcerative lesions in the skin (pyoderma gangrenosum), and acne. PSTPIP1 mutations generate other autoinflammatory diseases, like PSTPIP1-associated myeloid-related proteinemia inflammatory (PAMI) syndrome, previously known as hyperzincemia and hypercalprotectinemia [2, 3]. The autoinflammatory diseases caused by PSTPIP1 mutations are now collectively termed as PAID (PSTPIP1-associated inflammatory diseases). Moreover, the collection of diseases associated with PSTPIP1 mutations has expanded to include common variable immunodeficiency (CVID) [4].

**Fig. 1:**
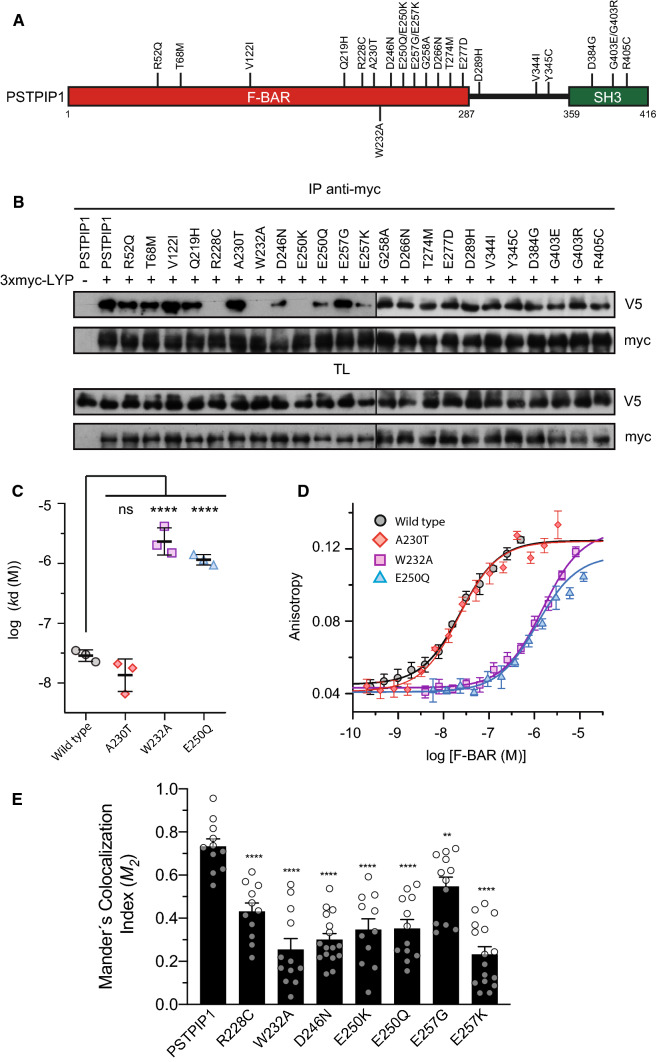
Interaction of LYP with of PSTPIP1 mutants associated to immune diseases. **A** Domain structure of PSTPIP1 with the mutations studied on this work. Mutations described in patients suffering from autoinflammatory diseases and CVID are indicated on top, while W232A mutation that is not related to disease is on the lower part. **B** HEK293 cells were transfected with PSTPIP1-V5 wild type and several mutants associated with autoinflammatory diseases, as indicated in the top of the panels, and with 3xmyc-LYP. Lysates were immunoprecipitated with myc antibody (Ab) and PSTPIP1 bound to LYP was detected by IB with Ab for the V5 epitope. Expression of the proteins was verified by IB in total lysates (TL) with the same Abs. **C** Dissociation constants (*k*_d_) of the interaction of the F-BAR domain of PSTPIP1, wild type and indicated mutants, with fluorescein-LYP-CTH. Data are means ± standard deviation (SD), *n* = 3 independent experiments, one shown in **D**. Statistical comparison to the *k*_d_ of the wild type F-BAR was analyzed using ANOVA followed by Dunnett’s test. ****p < 0.0001. **D** Representative binding isotherms of the F-BAR domain of PSTPIP1, wild type and mutants, to fluorescein-labeled LYP-CTH (2 nM), measured by fluorescence anisotropy. Data points are means ± SD of measurement replicates; lines represent the fitted binding curves. **E** HEK293 cells co-transfected with LYP-mCherry (red) and wild type or indicated mutants of PSTPIP1-eGFP (green). Manders’ colocalization indexes (M2) were calculated. Error bars represent SEM (*n* > 10). **p < 0.01; ****p < 0.0001, by Student’s *t* test

PSTPIP1 is an adaptor protein expressed in most immune cell lineages. It presents two structural domains in its sequence, an N-terminal Fer/CIP4 homology-Bin/Amphiphysin/Rvs (F-BAR) domain and a C-terminal SH3 domain, connected by a linker region (Fig. 1A). The function of F-BAR domains seems to be membrane shape coordination with the cytoskeleton in various cellular processes such as endocytosis and cell division [5–7]. F-BAR domains form homo-dimers with an elongated and slightly curved shape. Positively charged residues on the concave surface of F-BAR domains interact with the polar heads of the phospholipids in the cellular membrane [8, 9]. The F-BAR domain of PSTPIP1 binds to the PEST subfamily of protein tyrosine phosphatases (PTPs): PTPN12, also known as PTP-PEST, PTPN18, and the lymphoid phosphatase (LYP), encoded by the gene *PTPN22* [10]. These phosphatases bind to PSTPIP1 through a conserved motif in their C-terminus, thereby called C-terminal homology domain (CTH). LYP phosphatase is involved in autoimmune diseases with a significant inflammatory component, like arthritis or systemic lupus erythematosus [11]. The F-BAR domain of PSTPIP1 also binds pyrin, a sensor that activates the pyrin inflammasome in response to RhoA inactivation by bacterial toxins. Mutations in pyrin cause the autoinflammatory disease Familial Mediterranean Fever [12]. Additionally, the SH3 domain of PSTPIP1 interacts with ABL kinase [13], WASP [14], CD2 [15] and FasL [16], and may participate in the interaction with pyrin [17].

PSTPIP2, a paralog of PSTPIP1, presents mutations in mice that end up causing an autoinflammatory bone disease similar to chronic osteomyelitis [18, 19]. PSTPIP2 also has an F-BAR domain that shares 49% sequence identity with the F-BAR domain of PSTPIP1, followed by a short tail containing several tyrosines known to be phosphorylated, and not conserved in PSTPIP1 [20]. PSTPIP2 also binds to PEST phosphatases via its F-BAR domain [21, 22].

More than 20 missense mutations have been identified in PSTPIP1 in patients with autoinflammatory diseases [23], the majority of which are located in the F-BAR domain (Fig. 1A) [23–25]. Most of these mutations are currently uncharacterized, and thereby, it remains unknown how such a diverse array of changes in *PSTPIP1* gene, mostly inherited in a dominant fashion, alter PSTPIP1 function to cause the above referred immune diseases. Wise and co-workers identified *PSTPIP1* as the gene that originates PAPA syndrome and described the first mutations associated to this disease in PSTPIP1, A230T and E250Q [1], suggesting that the interaction of PSTPIP1 with PEST phosphatases could be critical to develop PAPA.

Thus, we studied the PSTPIP1/LYP interaction to gain a better understanding of the mechanism underlying PSTPIP1-related autoinflammatory diseases. We determined the previously unknown structure of the isolated F-BAR domain of PSTPIP1. Furthermore, we solved the structure of the F-BAR domain bound to the LYP CTH motif, which is the first high-resolution structure of an F-BAR domain bound to its ligand. This structure shows a new way of interacting Pro-rich peptides with proteins, in which the F-BAR dimer of PSTPIP1 binds to one molecule of the CTH peptide of LYP. Analysis of several mutations identified in patients show that only mutations R228C, E250Q, E250K, E257K, and D246N block the binding to LYP, and do not affect PSTPIP1 oligomerization. As a result, we conclude that disrupting the interaction between LYP and PSTPIP1 could play a key role in the mechanism underlying the autoinflammatory diseases caused by PSTPIP1 mutation.

## Materials and methods

### Antibodies and cell lines

The hemagglutinin (HA) monoclonal antibody (mAb) was from Covance (Berkely, CA, USA). The anti-V5 mAb was from Invitrogen (Carlsbad, CA, USA). The myc Ab (9E10) was from Santa Cruz Biotechnology Inc. (Santa Cruz, CA, USA). PSTPIP1 Ab was generated against the whole protein produced in bacteria [26]. The anti-LYP goat polyclonal Ab was from R&D Systems, Inc. (Minneapolis, MN, USA). The anti-GFP Ab was from eBioscience (San Diego, CA, USA). HEK293 were maintained at 37 °C in Dulbecco’s modified Eagle’s medium supplemented with 10% fetal bovine serum, 2 mM l-glutamine, 100 U/mL penicillin G, and 100 µg/mL streptomycin. Transient transfection of HEK293 cells was carried out using the calcium phosphate precipitation method [27].

### Plasmids and mutagenesis

PSTPIP1 (Uniprot O43586) wild type sequence in pcDNA3.1/V5-His and the mutants A230T and E250Q were a kind gift from Dr. Carol Wise. pEF3xmyc-LYP has been described previously [26]. Standard molecular biology techniques were used to generate the different constructs used in this study. Point mutations were created by PCR using the QuikChange method. All constructs and mutations were verified by nucleotide sequencing. For recombinant expression of PSTPIP1 in bacteria the cDNA coding for the region 1–289 of human PSTPIP1 was cloned in a modified version of the pET15b vector that codes for a fusion protein with an N-terminal poly-His tag and a site recognized by the tobacco etch virus (TEV) protease [28].

### Immunoprecipitation, SDS PAGE and immunoblotting

These procedures were done as reported before [29]. Briefly, cells were lysed in TNE lysis buffer: 20 mM Tris/HCl (pH 7.4), 150 mM NaCl, 5 mM EDTA containing 1% NP-40, 1 mM Na_3_VO_4_, 10 µg/mL aprotinin and leupeptin, and 1 mM PMSF, and clarified by centrifugation at 15,000 rpm for 10 min. The clarified lysates were preadsorbed on protein G-Sepharose and then incubated with Ab and protein G-Sepharose beads for 1 h. Immune complexes were washed three times in TNE buffer and suspended in SDS sample buffer. Proteins resolved by SDS-PAGE were transferred electrophoretically to nitrocellulose membranes, which were immunoblotted with optimal dilutions of specific Abs, followed by the appropriate anti-IgG-peroxidase-conjugate. Blots were developed by the enhanced chemiluminescence technique with Pierce ECL Western Blotting substrate (Thermo Scientific, Rockford IL) according to the manufacturer’s instructions.

### In vitro pull-down assays

Recombinant His-PSTPIP1 full-length produced in bacteria (2 μg) was combined with 2 μg of recombinant GST-CTH wild type or mutated in R799 or W805 to A, and 10 μL of glutathione sepharose beads (Cytiva, Marlborough, MA) in binding buffer: 5 mM Tris/HCl (pH 7.4), 50 mM NaCl, 0.1% NP-40. After incubation for 1 h at 4 °C, beads were washed three times in the same buffer and suspended in SDS sample buffer. The complexes were then processed for immunoblotting as explained above for the immunoprecipitation.

### Colocalization analysis

HEK 293 cells expressing LYP-mCherry wild type and PSTPIP1-eGFP wild type or indicated mutants were nuclei counterstained with the DNA binding dye DAPI. All images were captured with a Leica confocal system TCS SP5X inverted microscope with a HCS Plan Apo CS 63X/1.4 NA oil immersion lens. Leica Application Suite Advanced Fluorescence software was used for the capture, and ImageJ for image presentation.

Colocalization was analyzed with JACoP plugin in ImageJ. After background subtraction; negative pixel values were clipped to zero. Positive values were selected by Costes automatic thresholding, removing the bias of visual interpretation [30]. Colocalization index Manders’ *M*_1_ and *M*_2_, were calculated. Manders’ split coefficients are based on the Pearson’s correlation coefficient but avoid issues relating to absolute intensities of the signal, since they are normalized to total pixel intensity [31]. These coefficients vary from 0 (non- overlapping) to 1 (100% colocalization). The index *M*_2_ is defined as the percentage of above-background pixels from the second, red channel (LYP) that overlap above-background pixels from the first, green channel (PSTPIP1). This index is sensitive to changes in the background but not to differences in the intensity of overlapped pixels and is suitable to apply in images with a high and very clear signal to background ratio.

### Protein purification

Proteins were produced in the *Escherichia coli* strain BL21 (DE3). They were purified by nickel-chelating affinity chromatography, the His-tag was cleaved by digestion with TEV protease and removed as described [32].

Peptides of human LYP (Uniprot Q9Y2R2-1) corresponding to the region 787–807 (GFANRFSKPKGPRNPPPTWNI) were custom synthesized either labeled with fluorescein at the N-terminus (Thermo Scientific, Germany) or unlabeled (Genosphere Biotechnologies, France).

### Fluorescence-based binding assay

Binding of the F-BAR of PSTPIP1 to a peptide of the CTH of LYP labeled with fluorescein was analyzed by fluorescence anisotropy. Fluorescein-LYP-CTH in 20 mM Tris/HCl (pH 7.5), 150 mM NaCl, 0.1 mg/mL bovine serum albumin (BSA) were titrated with the F-BAR of PSTPIP1, wild type and point mutants. The fluorescence anisotropy was measured at 25ºC in a Fluoromax-3 spectrofluorometer (HORIBA-Jobin–Yvon) with Glan–Thompson polarizers using a 3 × 3 mm quartz cuvette. The samples were excited at 490 nm and the emission was collected at 520 nm. The apparent dissociation constant (*k*_d_) was estimated by fitting a one-to-one binding model as described [33] using the SigmaPlot program.

Alternatively, to determine the stoichiometry of the interaction, titrations were done under saturation conditions. Fluorescein-LYP-CTH at 1 μM, which was ~ 50 times above the *k*_d_ value, was titrated with PSTPIP1. Under these conditions, PSTPIP1 binds to the CTH in a linear fashion until all the CTH sites are occupied. The binding stoichiometry was estimated from the intersection of asymptotes of the ascending and plateau regions.

### Isothermal titration calorimetry (ITC)

ITC experiments were carried out at 25 °C using a VP-ITC system (MicroCal, Northampton, MA, USA). Solutions of the F-BAR of PSTPIP1 were loaded on the sample cell at a known concentration between 26.2 and 30.6 μM in 20 mM sodium phosphate (pH 7.5), 150 mM NaCl, 5% (v/v) dimethyl sulfoxide (DMSO). Solutions of the unlabeled peptide of the CTH of LYP, in the same buffer as the PSTPIP1 samples to avoid buffer mismatch and at known concentrations between 146.8 and 155.2 μM, were loaded in the injection syringe. Sample solutions were degassed and thermostated at 25 °C using a MicroCal ThermoVac sample station prior to use. Titrations were done by one initial injection of 3 μL followed by 27 sequential injections of 10 μL. A reference power of 15 μcal s^−1^, stirring speed of 307 rpm and 240 s spacing were selected. Heat exchange from the first injection was not used in the analysis. Data were analyzed using the Origin 7 ITC software package (MicroCal Software, Northampton, MA, USA), corrected by the heat of injection calculated from the basal heat remaining after saturation and confirmed by titration into buffer only as control. The single set of identical sites model was applied to obtain the binding enthalpy (∆*H*), entropy (∆*S*), stoichiometry (*N*), and association constant (*k*_a_ = 1/*k*d) using a nonlinear squares algorithm.

### Crystallization and structure determination of the G258A mutant of the F-BAR domain of PSTPIP1

Crystals of the F-BAR domain of PSTPIP1, residues 1–289, carrying the G258A mutation were obtained by hanging drop vapor diffusion at room temperature. A protein solution at 1.2 mg/mL in 10 mM Tris/HCl (pH 7.5), 150 mM NaCl, 0.5 mM dithiothreitol (DTT) was mixed with an equal volume of the crystallization solution 100 mM Bis–Tris-propane (pH 6.5), 15% (w/v) PEG 3350, 250 mM sodium citrate. Prior to data collection crystals were transferred to 100 mM Bis–Tris-propane (pH 6.5), 15% (w/v) PEG 3350, 250 mM sodium citrate, 18% glycerol and were cooled by immersion in liquid nitrogen. These and all other diffraction data were collected at 105 K on the I03 beamline of Diamond Light Source synchrotron (Didcot, UK). Diffraction intensities of this and other crystals (see below) were integrated, reduced, and converted into structure factor amplitudes with the programs XDS [34], Pointless [35], Aimless [36] and Staraniso [37] as implemented in the autoPROC pipeline [38].

Crystals belong to the space group P2_1_2_1_2_1_ (Table S1) and contain two PSTPIP1 molecules in the asymmetric unit (AU), which correspond to ~ 53% solvent content. Diffraction data was notably anisotropic. The lowest resolution limit was ~ 2.9 Å in the direction b* and the highest limits were 1.97 Å and 2.09 in the directions a* and c*, respectively. Staraniso was used to apply an anisotropic cut-off and correction of the intensity data, and to calculate the structure amplitudes using Bayesian estimation.

The structure was phased by molecular replacement (MR) using Phaser [39]. Initially, the MR was solved using a mixed-atom homology model built with SCWRL [40] using as template the crystal structure of Hof1p F-BAR domain (PDB ID 4WPE), which shares 14% sequence identity with PSTPIP1. Starting from this MR solution the structure was refined with Phenix Refine [41] against the anisotropically corrected data, alternated with manual model building in Coot [42]. Yet, it was not possible to improve the refinement of this model; the free R factor was ~ 47%. At this point, we perform normal mode analysis (NMA) on the partially refined structure using the elNemo sever, a web interface to do elastic network model-based NMA [43]. Eleven conformations that represent global motions corresponding to the lowest-frequency mode (an amplitude perturbation in the direction of a single normal mode of 500 was applied by using a 50 step size) were generated. Each of these conformations was used as search model to phase again the structure by MR. The model with best MR solution scores was automatically rebuilt using Phenix Autobuild [44]. The resulting model was refined as described above. Torsion angle non-crystallographic symmetry (NCS) restraints were used. Two TLS (Translation-Libration-Screw-rotation) groups, one per molecule, were refined. The refined model includes residues 5–289 of each of the two PSTPIP1 molecules in the AU, and 310 water molecules. The structure has 99.3% residues in most favored regions of the Ramachandran plot and the remaining in additionally allowed regions (Table S1).

### Crystallization and structure determination of the wild type F-BAR domain of PSTPIP1

Crystals of the wild type F-BAR domain of PSTPIP1 were obtained similarly as for the G258A mutant. Briefly, a protein solution at 0.5 mg/mL in 10 mM Tris/HCl (pH 7.5), 100 mM NaCl, 0.5 mM DTT was mixed with an equal volume of 100 mM Bis–Tris-propane (pH 6.0), 17% (w/v) PEG 3350, 150 mM sodium citrate and was equilibrated against the latter. Crystals were flash cooled in liquid nitrogen using 19% glycerol as cryoprotectant. Data were collected and processed as described above. A high multiplicity dataset was obtained by combining two sets of 2400 and 1200 images, 0.15° oscillation per image, collected on two separate regions of the same crystal.

Crystals were isomorphic to those of the G258A mutant. The diffraction was strongly anisotropic with approximate resolution limits of 2.12, 4.32, and 2.17 Å in the directions a*, b* and c*, respectively. The structure was refined against the anisotropically corrected data as for the G258A mutant, using the structure of the mutant as the starting model. The refined structure had a free R factor of 24.1% (Table S1).

### Crystallization and structure determination of the F-BAR domain of PSTPIP1 in complex with LYP

To obtain crystals of the PSTPIP1/LYP complex, the peptide of the CTH of LYP (residues 787–807) was soaked into preformed crystals of the wild type F-BAR of PSTPIP1. A 4.2 mM solution of the peptide in 100 mM Bis–Tris-propane (pH 6.0), 20% PEG 3350, 200 mM sodium citrate was added to a crystallization drop with the PSTPIP1 crystals. After 1 h incubation at room temperature the crystals were briefly transferred into a cryoprotectant solution containing 20% glycerol and were flashed cooled. Diffraction data were collected and processed as for the previous crystals. A dataset was obtained by combining three sets of 1800 images, 0.1° oscillation per image, measured in three regions of a crystal. Crystals were isomorphic to those of the wild type and G258A mutant of PSTPIP1. The diffraction was also highly anisotropic; the approximate resolution limits were 4.05 Å in the direction b* and ~ 2.1 Å in directions a* and c*.

The structure of the complex was refined against the anisotropically corrected data as for the isolated PSTPIP1 structures. The structure of the wild type F-BAR was used as starting model. The LYP segment was built using Coot and 2*mFobs-DFcalc* maps. Refinement converged to a free R factor of 24.1%. The refined model (Table S1) includes residues 5–289 and 3–289 of the two PSTPIP1 molecules in the AU, respectively, residues 793–806 of LYP, 162 waters, and two molecules of glycerol.

### Sequence analysis

Sequences similar to the PEST phosphatase-binding site of PSTPIP1/2 were identified using HMMER (v3.3) [45]. A hidden Markov model profile build using the regions 225–262 of PSTPIP1 and PSTPIP2 was used to search the UniProtKB/Swiss-Prot reference proteomes (Release 2020/01) [46]. This identified similarity with regions of *Schizosaccharomyces pombe* Cdc15 (Uniprot entry Q09822, residues 234–271) and Imp2 (Uniprot Q10199, residues 221–258).

### Structure analysis

Protein–protein contacts were analyzed with the PISA server [47]. Calculation of electrostatic potential was performed with the program APBS [48]. Molecular figures were prepared with PyMol [49]. The Cdc15/Cdc12 complex was modeled using the structure of LYP bound to PSTPIP1 as template. The PSTPIP1/LYP complex was superimposed onto the structure of Cdc15 by fitting the α4 helices of the F-BAR dimers. Next, the residues in LYP were mutated to the equivalent residues in Cdc12; the side chains that were changed were modeled as energetically favorable conformations.

## Results

### Effect of PSTPIP1 mutations associated to PAPA on the interaction with LYP and other PEST phosphatases

PSTPIP1 mutations A230T and E250Q, which were initially identified as the cause of PAPA autoinflammatory disease, restricted PSTPIP1 binding to the tyrosine phosphatase PTP-PEST [1], implying that the interaction between PSTPIP1 and PEST phosphatases is important in the etiology of PAPA autoinflammatory syndrome. Furthermore, since the discovery of A230T and E250Q mutations in PSTPIP1 in 2002, over 20 missense mutations have been linked to autoinflammatory diseases (Fig. 1A) [23]. Given that LYP is involved in inflammatory autoimmune diseases such as arthritis and lupus, we wanted to see how PSTPIP1 mutations affected its interaction with LYP. Toward this end, transiently transfected HEK293 cells with full-length PSTPIP1 and LYP were used to assess, by immunoprecipitation, the effect on PSTPIP1/LYP interaction of an ample set of mutations identified in PSTPIP1 in patients with autoinflammatory diseases. The mutation W232A, which prevents PSTPIP1 from interacting with PTP-PEST [50] and LYP [26], but is unrelated to illness, was also included. Our results showed that mutations R228C, D246N, E250Q, E250K and E257K, in addition to W232A, reduced the interaction with LYP (Fig. 1B). To exclude any possible indirect effect of the inhibitory mutations on the interaction between PSTPIP1 and LYP in HEK293 cells, we also analyzed their direct binding in vitro. Using the purified F-BAR domain of PSTPIP1 (residues 1–289) and a synthetic peptide of the CTH of LYP (residues 787–807) labeled with fluorescein, we measured the affinity of the interaction (Fig. 1C, D). The wild type F-BAR and the A230T mutant bound to LYP with similar affinity; their apparent dissociation constants (*k*_d_) were ~ 20 nM. On the other hand, the binding affinities of the mutants E250Q and W232A were drastically lower (~ 60-fold), in agreement with the loss of interaction observed in the immunoprecipitation experiments.

As our results partially differed from those reported previously for PTP-PEST [1], we tested the interaction of this phosphatase and PTPN18 with PSTPIP1 mutants to see whether this discrepancy was merely due to the phosphatase used in the assay (Supplemental Fig. S1). In these experiments, the effect of the assayed point mutations in PSTPIP1 on the interaction with PTP-PEST and PTPN18 was identical to that observed for the interaction with LYP.

Collectively, our data suggest that the PEST phosphatases LYP, PTP-PEST and PTPN18 interact with PSTPIP1 in a similar way, and that the amino acids R228, W232, D246, E250 and E257 of PSTPIP1 are important for the association with the PEST phosphatases, indicating that a cluster of residues in PSTPIP1 F-BAR domain is critical for the interaction with these phosphatases.

Given that PSTPIP1 is located in the membrane through the F-BAR domain, we tested whether the mutations that alter the interaction with LYP, affected its recruitment to the membrane. We transfected HEK293 cells with full-length LYP-mCherry and PSTPIP1-eGFP wild type or mutated in amino acids that make contact with LYP. Colocalization between LYP and PSTP1 constructs was studied by confocal microscopy using the Manders’ colocalization index M2 [31] (Fig. 1E and Supplemental Fig. S2). These data indicate that PSTPIP1 mutations that reduce the interaction with LYP, also diminished its recruitment to the membrane, what would affect accessibility of LYP to its substrates and subsequently its dephosphorylation by LYP.

### Structure of the F-BAR domain of PSTPIP1

To better understand the molecular basis of the physiological function of PSTPIP1 in the immune system, and its role in autoinflammatory diseases, we elucidated the 3D structure of the F-BAR domain (residues 1–289) using X-ray crystallography. Initially, we solved the structure of the G258A mutant, and subsequently that of the wild type protein. The two structures were almost identical. After superimposition, the root mean square deviation (rmsd) between all the equivalent Cα atoms was 0.275 Ǻ, which was similar to the estimated errors of the coordinates of the structures (0.23–0.24 Ǻ). Since the G258A structure was refined to a higher resolution, hereafter, we refer to it.

**Fig. 2:**
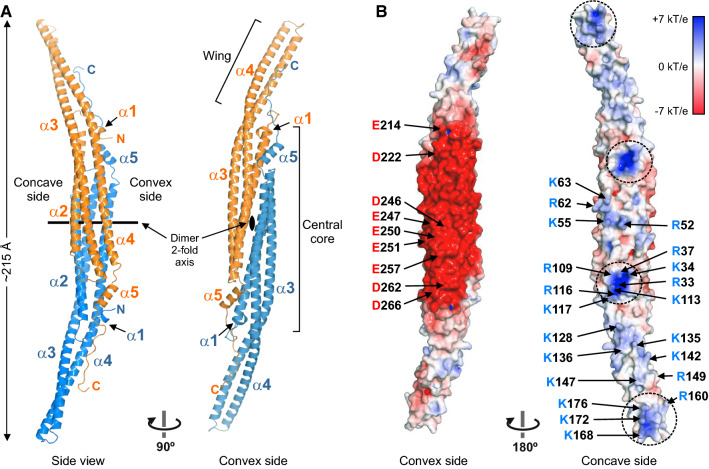
**Structure of the F-BAR domain of PSTPIP1.** (**A**) Two orthogonal views of a ribbon representation of the structure of PSTPIP1. Protomers of the dimer are colored in orange and blue, respectively. (**B**) Surface representation of the F-BAR dimer colored by the electrostatic potential. Clusters of basic residues in the concave surface are encircled with dashed lines. Acidic and basic residues on the surface are labeled in one of the protomers

The asymmetric unit of the crystal contains two molecules of PSTPIP1 that form the characteristic elongated crescent-shape of F-BAR dimers (Fig. 2A) [50]. Each protomer consists of five α-helices, with the longer helices, α2, α3, and α4, forming a helical bundle. The dimerization interface is mainly formed by α2, α4, and the shorter helix, α5. Part of helices α3 and α4 protrude from the central dimerization core region forming the so-called wings, which are bent ~ 30° with respect to the longitudinal axis of the central region when viewed from the concave or convex sides, resulting in a tilted shape.

The F-BAR dimer of PSTPIP1 bears structural resemblance to the F-BAR of Imp2 of *Schizosaccharomyces pombe* (PDB ID 5C1F, 27% sequence identity) [51]; after superimposition, the rmsd for 556 Cα atoms was 2.4 Ǻ. PSTPIP1 is also very similar to the F-BAR domains of Hof1p of *Saccharomyces cerevisiae* (PDB ID 4WPE, rmsd 2.7 Ǻ for 550 Cα atoms, 15% sequence identity) [52], Cdc15 of *S. pombe* (PDB ID 6XJ1, rmsd 2.9 Ǻ for 532 Cα atoms, 20% sequence identity) [53], and the human Cdc42-interactin protein 4 (CIP4) (PDB ID 2EFK, rmsd 3.1 Ǻ for 534 Cα atoms, 20% sequence identity) [8]. The minor differences between PSTPIP1 and these F-BAR structures are most noticeable in the wings (Supplemental Fig. S3). The curvature of the F-BAR dimer of PSTPIP1 is most similar to that of Cdc15 and CIP4, and it is only slightly straighter than the F-BAR domains of Imp2 and Hof1p. This is supported by the fact that the liposome tubules induced by PSTPIP1 (diameter 60–80 nm) [54] and the tubules formed by Imp2 [51] and CIP4 [55] present a comparable diameter.

The concave side of the F-BAR dimer is overall electropositive and contains two clusters of basic residues in each protomer, one in the core region, and the other near the tip of the wing (Fig. 2B). Similar electro-positive patches are observed in the F-BAR domains of Imp2 [51], FCHo2 (PDB ID 2V0O) [56], the Gem-interacting protein (PDB ID 3QWE), and the RhoGAP Rgd1p (PDB ID 4WPC) [52] (Supplemental Fig. S4); which are important for binding to membranes with anionic groups.

On the other hand, the central area of the convex surface has strong electronegative character due to the presence of multiple acidic residues. Large acidic surfaces are observed in the F-BAR domains of other proteins, such as Cdc15, growth arrest-specific 7 (GAS7), and formin-binding protein 17 (FBP17), but are absent in several F-BAR domains (Supplemental Fig. S4). The residues of PSTPIP1 critical for binding to PEST phosphatases (see above) are located in this electronegative area, supporting the notion that the acidic convex area is important for PSTPIP1 function.

### Structure of the F-BAR domain of PSTPIP1 in complex with LYP

To visualize directly the PSTPIP1/LYP interaction we produced crystals of the F-BAR domain of PSTPIP1 in complex with a peptide of the CTH of LYP, residues 787–807. An additional continuous electron density, adjacent to PSTPIP1, was assigned to residues 793–806 of LYP (Supplemental Fig. S5). No electron density was observed for the first six residues of the CTH, suggesting that they do not engage in specific contacts with PSTPIP1.

**Fig. 3:**
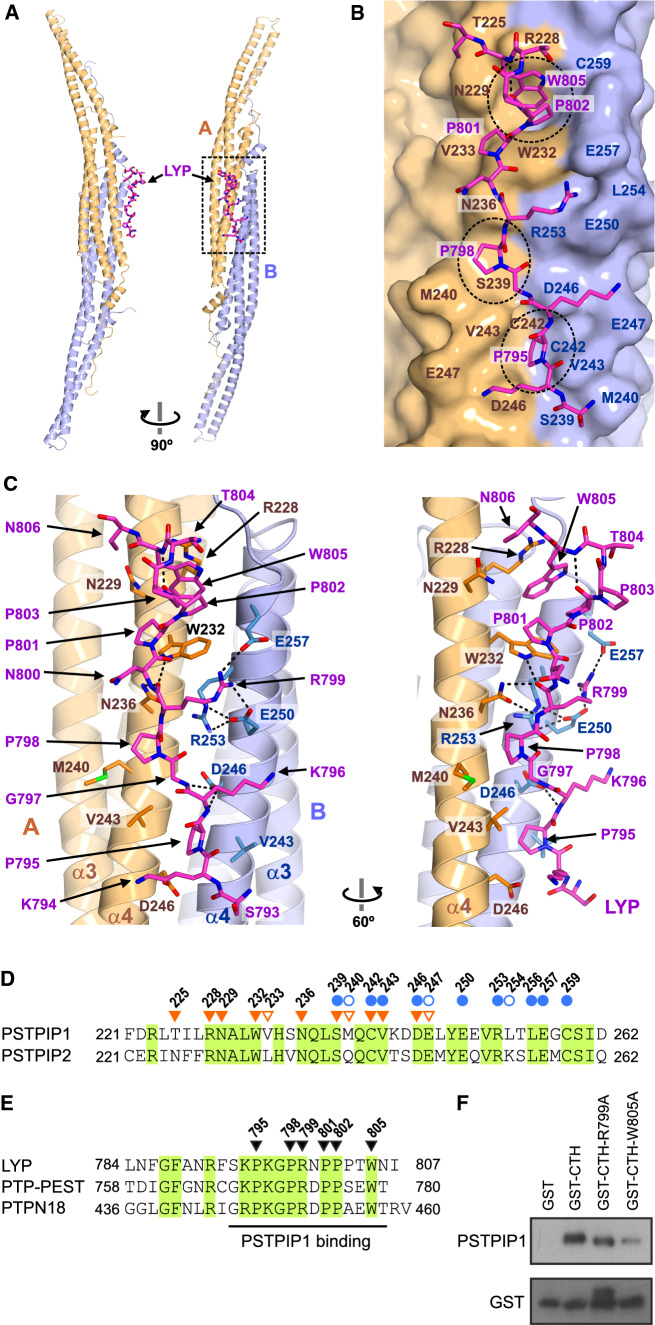
Structural determinants of the PSTPIP1/LYP interaction. **A** Orthogonal views of the overall structure of the complex. The two protomers of the PSTPIP1 dimer, A (orange) and B (blue), and LYP (magenta) are colored similarly throughout the figure. **B** Interaction of LYP, shown as sticks, with PSTPIP1, shown as surface. Residues of PSTPIP1 that participate in the interface are labeled. The three hydrophobic pockets of the binding site are highlighted by dashed-line circles. **C** Two views showing contacts between LYP and PSTPIP1. H-bonds and salt bridges are shown as dashed lines. **D** Sequence alignment of the regions of human PSTPIP1 and PSTPIP2 (Uniprot Q9H939) that contain the LYP-binding site. Residues from protomers A and B, which form the interface, are indicated by inverted orange triangles and blue circles, respectively. Residues whose solvent exposed surface was buried ≤ 20% by LYP are indicated by open symbols. Conserved residues are shown in green boxes. **E** Multiple sequence alignment of the CTH regions of phosphatases LYP, PTP-PEST (Uniprot Q05209) and PTPN18 (Uniprot Q99952). Residues of LYP that make specific contacts with PSTPIP1 are indicated by inverted triangles. Conserved residues are shown in green boxes. (F) His-PSTPIP1 full- length recombinant protein was subjected to pulldown assays with the indicated CTH peptide fused to GST. The presence of PSTPIP1 in the precipitates was visualized by IB with antibody against PSTPIP1, and GST–peptides were detected with an antibody against GST

A single copy of LYP binds to the convex surface of the PSTPIP1 dimer at the rim of the dimerization interface formed by helices α4 of the two protomers (Fig. 3A). LYP buries ~ 625 Ǻ^2^ (2.1%) of the surface area of the PSTPIP1 dimer and establishes distinct contacts with each of the PSTPIP1 protomers; hereafter we refer to them as A and B. The LYP-binding site is characterized by three hydrophobic pockets aligned on the surface of PSTPIP1 (Fig. 3B, C). The first segment of LYP, residues 793–800, binds in an extended conformation. P795 docks in a first pocket that is delimited by V243 and D246 of each protomer. D246 of protomer B also forms hydrogen bonds (H-bond) with the backbone amide groups of K796 and G797 of LYP. P798 is cradled in a shallow second pocket. The side chain of the adjacent R799 makes ionic contacts with E250 and E257 of the protomer B; in addition, the main-chain amide and carbonyl of R799 make H-bonds with the side chain of N236 of protomer A. The C-terminal part of LYP (801–805) interacts with a deeper pocket formed between R228 and W232 of protomer A. P801 and P802 adopt a poly-proline II helix conformation; the ring of P801 contacts W232 in parallel. P802-W805 form a type I β-turn stabilized by a H-bond between the carbonyl of P802 and the amide group of W805. This creates a stacking of the rings of P802 and W805. In turn, W805 makes a stacking cation-π interaction with R228 of PSTPIP1. P803 and T804 of LYP are exposed to the solvent and do not contact PSTPIP1. Nonetheless, they might contribute to the stabilization of the LYP backbone conformation, because Pro and Thr frequently appear in these positions of type I β-turns [57]. The side chains of K794 and K796 of LYP do not engage in specific contacts with PSTPIP1. Nevertheless, K794, K796, and R791 (the latter is disordered in the structure) might favor the association of LYP with PSTPIP1 via electrostatic complementarity with the acidic surface around the binding site. Finally, the conformation of the PSTPIP1 residues involved in LYP binding is very similar in the free and bound structures (Supplemental Fig. S6). The pre-organized conformation of the LYP-binding site might favor the interaction.

The residues of PSTPIP1 that participate in the LYP-binding area are conserved in PSTPIP2 (Fig. 3D), with the exception of V233, M240, and L254, which are in the periphery of the interface and do not make specific contacts with LYP. The LYP-binding interface is more conserved (78% identity between PSTPIP1 and PSTPIP2), than the rest of their F-BAR domains (49% identity), suggesting functional conservation. On the other hand, the residues of LYP that make the key interactions with PSTPIP1 are conserved in PTP-PEST and PTPN18 (Fig. 3E). Collectively, the conservation of the interaction interfaces supports the notion that the structure of the PSTPIP1/LYP complex represents a general model for the binding of the PEST phosphatases LYP, PTP-PEST, and PTPN18, to PSTPIP1 and PSTPIP2.

To verify experimentally the LYP residues critical for the interaction we mutated in the CTH sequence amino acids R799 and W805 to Ala. In vitro binding assays were conducted with His-PSTPIP1 recombinant full-length protein obtained from bacteria and GST-CTH proteins. Mutations R799A and W805A reduced the interaction with PSTPIP1, in agreement with structural data (Fig. 3F).

We also analyzed whether the characteristics of the LYP-binding site of PSTPIP1/2 are present in other F-BAR domains. Using a profile hidden Markov model method, sequence similarity was detected between the α4 segments of PSTPIP1/2 and the *S. pombe* proteins Cdc15 and Imp2. The sequence identity in the α4 between PSTPIP1 and Cdc15 and Imp2 is 59 and 42%, respectively; while the overall identity is lower (27 and 32%). The key residues of the LYP binding site of PSTPIP1 are conserved in Cdc15 and Imp2 (Supplemental Fig. S7A). The F-BAR domains of Imp2 [51] and Cdc15 [53] have similar pockets to those in the LYP binding site of PSTPIP1 (Supplemental Fig. S7B–C). The F-BAR of Cdc15 binds to a short sequence near the N-terminus of the formin Cdc12 (24-SARRTIGPRAPKS-36) [58], which shares five conserved residues with the core of the CTH of the PEST phosphatases (Supplemental Fig. S7D). We modeled the Cdc15/Cdc12 interaction using the F-BAR structure of Cdc15 and the PSTPIP1/LYP complex (Supplemental Fig. S7C). In this model, Cdc15 residues D255 and E259 make polar contacts with Cdc12 and E256 is part of one of the binding pockets, which is in agreement with the loss of Cdc12 binding when either of these acidic residues are replaced by alanines [53]. The model also predicts docking of Cdc12 residues P31 and P34 in two pockets of Cdc15; which is consistent with the deleterious effect of the P31A substitution in Cdc12 on its binding to Cdc15 [58]. Finally, the Cdc15 binding motif of Cdc12 contains multiple basic residues that complement the electronegative character of the Cdc15 convex surface (Supplemental Fig. S4). In conclusion, the F-BAR domains of Cdc15 and PSTPIP1 appear to bind to ligands containing Pro-rich motifs in a similar manner.

### Only one molecule of LYP binds to each PSTPIP1 dimer

The first pocket of the LYP-binding site lies in the two-fold rotation axis that relates the two protomers of the PSTPIP1 dimer. Thus, only one molecule of LYP can bind to the PSTPIP1 dimer at any time. In the structure, LYP only appears bound in one orientation. This is caused by differences in the environment of the two sites in the crystal lattice; binding to one of the sites is hampered by steric clashes with a neighbouring PSTPIP1 molecule of the crystal (Supplemental Fig. S8).

**Fig. 4:**
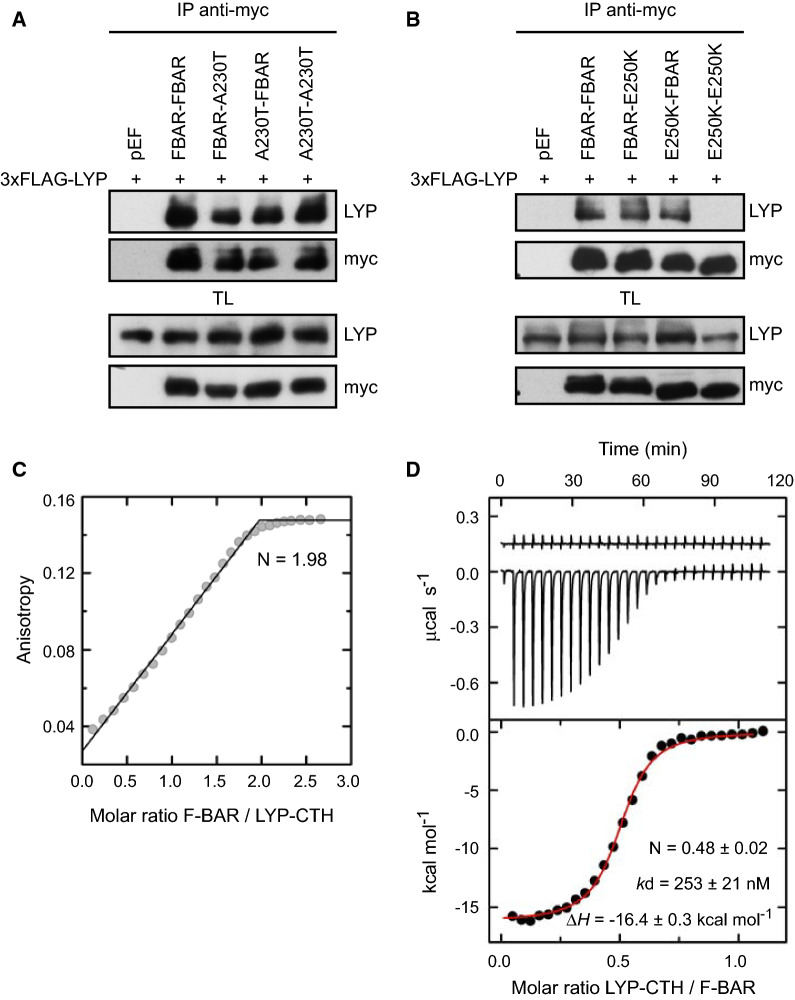
Interaction of LYP with F-BAR dimers. **A**, **B** HEK293 cells were transfected with PSTPIP1 F-BAR dimers in 3xmyc plasmid, as indicated in the top of the panels, and with 3xFLAG-LYP. Lysates were immunoprecipitated with myc Ab and LYP bound to PSTPIP1 was detected by IB with Ab for LYP. Expression of the proteins was verified by IB in total lysates (TL) with the same Abs. **C** Determination of the interaction stoichiometry by titration of fluorescein-LYP-CTH (1 µM) with the F-BAR of PSTPIP1 (residues 1–289) using fluorescence anisotropy. The stoichiometry (*N*) was determined from the intersection of asymptotic lines in the linear ascending and saturation (i.e. plateau) regions. **D** ITC analysis of LYP binding to PSTPIP1. Upper panel, heat exchange of the dilution of the unlabeled LYP-CTH peptide in buffer 20 mM sodium phosphate (pH 7.5), 150 mM NaCl, 5% (v/v) DMSO (upper trace) and representative thermogram of the binding of LYP-CTH to the F-BAR of PSTPIP1 (lower trace). Lower panel, LYP binding isotherm to PSTPIP1. The red line is the fit of a one binding site model to the heat changes of the interaction after subtracting the heat change of the dilution of LYP in buffer. The reported values of *N*, *k*_d_, and binding enthalpy (Δ*H*) are the means ± standard deviations of three independent experiments

To assess whether the binding of one molecule of LYP to a dimer of PSTPIP1 observed in the crystal represents the actual stoichiometry of the complex, we tested by immunoprecipitation the interaction of LYP full-length with a single-chain pseudo-dimer of the F-BAR domain. To this end, we created a construct with two copies of the F-BAR domain in tandem in the same polypeptide. It should be noted that the C-terminus of each protomer is adjacent to the N-terminus of the companion protomer in the F-BAR dimer. We created this chimeric protein with either PSTPIP1 wild type or mutants A230T and E250K. Mutations were introduced independently in each one of the F-BAR copies or simultaneously in both copies. LYP bound to the wild type tandem dimer, to the single and double A230T dimers (Fig. 4A), and to any of the heterodimers carrying the E250K mutation in only one copy of the F-BAR (Fig. 4B). On the other hand, binding to LYP was lost when the tandem dimers carried the E250K mutation in both F-BAR domains (Fig. 4B). These results are in agreement with the crystal structure, in which only E250 from one of the protomers contacts LYP.

We also obtained independent estimations of the stoichiometry (*N*) of the complex using two orthogonal methods. First, analysis by fluorescence anisotropy of the binding of the F-BAR to fluorescein-LYP-CTH under saturation conditions yielded a binding ratio of two molecules of PSTPIP1 for each LYP-CTH (Fig. 4C). Second, we analyzed the binding of unlabeled LYP-CTH to the F-BAR of PSTPIP1 using isothermal titration calorimetry (ITC), which revealed a N of 0.48 ± 0.02 molecules of LYP-CTH for each PSTPIP1 (Fig. 4D and Table S2). The binding affinity determined by ITC (*k*_d_ = 253 ± 21 nM) was lower than in the fluorescence assays (Fig. 1C, D); these differences could be due to a positive contribution of the fluorescein probe and to the slightly different experimental conditions in each type of experiment.

Collectively, the structural and biochemical data support that each dimer of PSTPIP1 binds only one molecule of LYP. In the same way, one molecule of Cdc12 binds to each F-BAR dimer of Cdc15 [53]. Our model of the Cdc15/Cdc12 complex based on the PSTPIP1/LYP structure (see above) provides the structural basis for the stoichiometry of this interaction. Cdc12 occupies the pocket in the two-fold axis of the F-BAR dimer, hampering the binding of a second Cdc12 molecule, as LYP does in PSTPIP1.

### Rationalization of pathogenic mutations of PSTPIP1

**Fig. 5:**
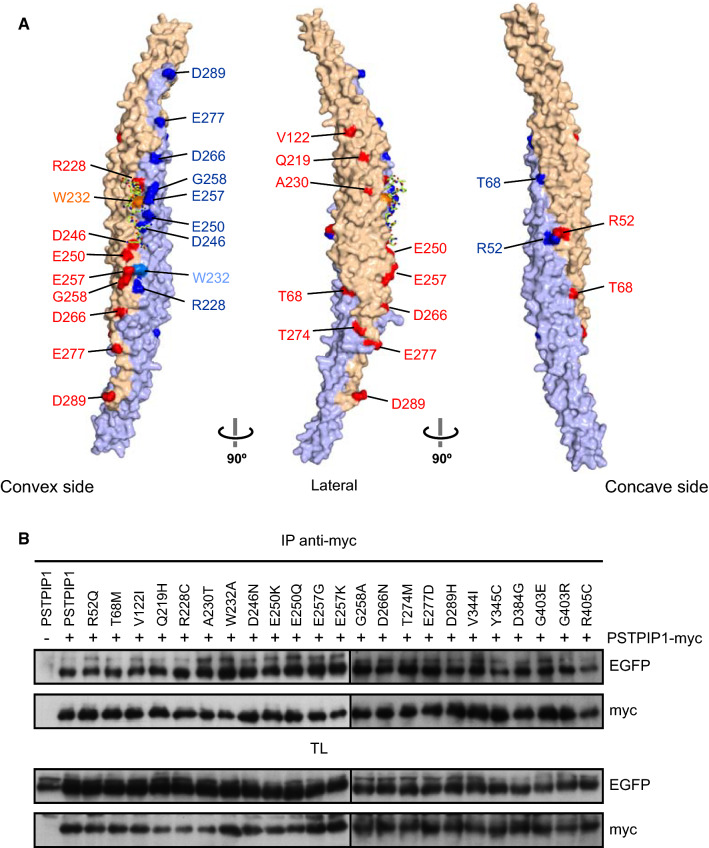
Pathogenic mutations of PSTPIP1. **A** Surface representation of the structure of PSTPIP1. Residues mutated in autoimmune diseases are shown in red and blue for each protomer of the F-BAR dimer, respectively. The position of W232 is also indicated. **B** HEK293 cells were transfected with several PSTPIP1 mutants fused to EGFP, as indicated in the top of the panels, and with PSTPIP1-myc. Lysates were immunoprecipitated with anti-myc Ab and the different mutants of PSTPIP1-EGFP precipitated with PSTPIP1-myc were detected by IB with anti-GFP Ab. Expression of the proteins was verified by IB in total lysates (TL) with the same Abs

More than 20 missense mutations have been described in PSTPIP1, among which 16 are located in the F-BAR domain (Fig. 5A). The only mutations found on the LYP-binding interface are R228C, D246N, E250Q, E250K, E257G, and E257K (Fig. 3B), in line with the loss of the interaction observed between LYP and PSTPIP1 carrying any of these mutations, except E257G (Fig. 1). R228C abolishes binding of LYP by removing the staking interaction with W805 of LYP. The reduced binding of LYP to PSTPIP1-D246N is likely to be caused by the alteration of the H-bonds established by D246 with the backbone of LYP and with the side chain of S239 in the companion protomer of the F-BAR dimer. The carboxylate of D246 is a double H-bond acceptor; but the carboxamide of the asparagine has donor and acceptor groups. The deleterious effects of the mutations E250Q and E250K are explained by the loss of a salt bridge with LYP-R799 and by the probable distortion of the binding interface due to the disruption of an intramolecular salt bridge with R253 of PSTPIP1. E257 of PSTPIP1 makes a second salt bridge with R799 of LYP. The mutation E257K abolishes almost completely the binding to LYP, but E257G has a smaller impact on this interaction, suggesting that the salt bridge between PSTPIP1-E257 and LYP-R799 is not essential for binding and that loss of binding in E257K might involve electrostatic repulsion with the CTH of LYP, which contains three positively charged residues.

Mutations D266N, E277D and D279H, affect acidic residues that are exposed to the solvent on the convex surface but do not make contacts with LYP (Fig. 5A), and those mutations do not interfere with the interaction. The same is valid for mutations T68M, V122I, G258A and T274M. On the other hand, the loss of a positively charged group in R52Q could affect the interaction of PSTPIP1 with the membrane, as R52 is exposed to the solvent on the concave surface of the F-BAR domain (Fig. 5A and Supplemental Fig. S9A). Finally, A230T mutation deserves some attention as this change is found in almost 25% of the patients with immune diseases [2]. This residue is located in the α4 helix, facing the core of the F-BAR helical bundle. Yet, a threonine in this position would not distort the structure and would not affect the nearby LYP-binding site (Supplemental Fig. S9B), in keeping with the lack of a noticeable effect of A230T on the binding of LYP. Altogether, only missense mutations in residues that participate directly in the PEST-binding site compromise PSTPIP1-LYP interaction.

Several pathogenic mutations affect residues that are located at or near the dimerization interface, such as R52, T68, R228, W232, D246, and T274. This prompted us to investigate if any of these or other mutations could affect the oligomerization of PSTPIP1. Self-association was tested by transient transfection of HEK293 cells with PSTPIP1-myc and PSTPIP1-EGFP, either wild type or carrying different mutations. Immunoprecipitation of PSTPIP1 with a myc antibody, led to co-precipitation of all the GFP-tagged mutants tested without significant differences (Fig. 5B). In summary, these mutations do not affect the oligomerization of the F-BAR domain of PSTPIP1.

## Discussion

In this work, we have studied the interaction of PSTPIP1 with LYP, due to its relevance for autoinflammatory diseases. Structural and functional analysis revealed that one copy of the Pro-rich CTH region of LYP binds, with high affinity, to one F-BAR dimer. LYP sequence recognition by PSTPIP1 relies on three hydrophobic pockets found in the middle of an acidic surface on the convex side of the F-BAR dimer, at the rim of the dimerization interface. Several prolines of the CTH play key roles in the interaction. P795, P798, P801, and P802 dock in the PSTPIP1 pockets, and P802 and P803 contribute to the correct orientation of W805 into the third pocket. In addition, the interaction is strengthened by basic residues of LYP, through the formation of ionic bridges and, possibly, via long range electrostatic contributions. The determinants of PSTPIP1/LYP recognition are conserved in PSTPIP2 and in the CTH domains of the PEST phosphatases PTP-PEST and PTPN18.

The main features of the PEST-binding site of PSTPIP1/2 are present in the F-BAR domains of the yeast proteins Imp2 and Cdc15. The Cdc15-binding site of Cdc12 has sequence similarity to the CTH of PESTs, suggesting that Cdc12 binds to Cdc15 in a similar manner to the PSTPIP1/LYP interaction. While no specific interaction of the F-BAR of Imp2 has been described so far, we hypothesize that it may also recognize basic Pro-rich ligands in a similar fashion. In summary, the PSTPIP1/LYP complex defines a novel mechanism of recognition of basic Pro-rich motifs by F-BAR domains that is different from the interaction modes of other Pro-rich-binding domains, such as SH3, WW, UEV, or EVH1 domains [59].

The structure of the PSTPIP1/LYP complex is the first high-resolution structural description of a protein–protein interaction by an F-BAR domain. Other F-BAR domains also mediate heterologous protein–protein interactions. For example, the PACSIN2 F-BAR domain binds to caveolin-1 [60, 61], polycystin-1 [62], filamin A [63, 64], actin [65], and to the nonstructural protein 5A (NS5A) of hepatitis C virus [66]. Hof1 F-BAR domain binds, through the tips of the F-BAR dimer, to the formin homology 2 (FH2) domain of Bnr1 formin protein [67]; and the F-BAR domain of Rag7 binds to the coiled-coil protein Rng10 during cytokinesis [68]. Yet, high-resolution structures of protein complexes mediated by F-BAR domains remain unknown and the interaction sites are mostly unmapped. It is possible that the area around helix α4 and the inter-dimer rim in the convex side of other F-BAR domains also mediate the interaction with additional proteins. The architecture of the PSTPIP1/LYP complex differs from the interactions stablish by the N-BAR of arfaptin with Rac1 via the concave surface of the N-BAR domain [69], and with Arl1 through the lateral regions of the N-BAR domain [70].

Here, we have analyzed the impact of a large set of PSTPIP1 mutations, distributed through the different domains of PSTPIP1, on its interaction with LYP, and on its oligomerization. The disease-linked mutations occur mainly on surface-exposed amino acids, suggesting they could affect the oligomerization of PSTPIP1 or its interaction with other molecules. However, only a small group of mutations clustered in the α4 helix of the F-BAR domain compromise the PSTPIP1/LYP interaction. On the other hand, none of the mutations tested in this study affect PSTPIP1 oligomerization. The mutations that alter the binding to LYP and other PEST phosphatases: R228C, D246N, E250Q, E250K and E257K, although few in number, represent around 50% of the patients with immune diseases associated to PSTPIP1 mutations [4, 71]. Notably, these mutations have been identified in patients with different diseases, R228C has been described in a patient with CVID, D246N and E250Q are associated with PAPA, and E250K and E257K cause PAMI, a more severe autoinflammatory disease than PAPA. Our data clearly show that the interaction between LYP and PSTPIP1 is exclusively affected by mutations in the residues involved in this interaction. Therefore, other mutations, in the case of being pathogenic, would act by affecting additional interactions or functions of PSTPIP1.

The mutation A230T appears in 25% of the patients with PSTPIP1 associated diseases [2]. However, A230 makes no contact with the CTH peptide and accordingly, this mutation has no effect on LYP interaction (Fig. 1B-D), as we reported previously [26]. This residue is highly variable among PSTPIP1 orthologues, suggesting that changes in this position are permissible. On the other hand, A230T has been reported to increase the interaction of PSTPIP1 with pyrin [17], which was hypothesized to be caused by a decrease in the interaction with PTP-PEST. In agreement with our data, the referred increase in the interaction between PSTPIP1 and pyrin caused by A230T mutation should be produced by a different mechanism. In this sense, introduction of A230T mutation in mice does not recapitulate the inflammatory disease observed in patients [72].

The PSTPIP1 F-BAR domain binds to membranes through the electro-positive concave surface [9], while LYP binds to the convex surface. Thus, PSTPIP1 may engage simultaneously in both interactions leading to the recruitment of PEST phosphatases to the plasma membrane. The high affinity interaction between PSTPIP1 and LYP suggests they form a stable complex that serves to target the substrates of the phosphatase, either recruited directly through the interaction with the SH3 domain of PSTPIP1, or indirectly by proximity to PSTPIP1, likely in the plasma membrane. Proteins known to bind to the SH3 domain of PSTPIP1, like WASP and ABL, have been suggested to be substrates of PEST phosphatases [13, 73]. ABL is also critical for phosphorylation of PSTPIP1 in Y345 [13]. Mutations in PSTPIP1 that reduce the interaction with PEST phosphatases will increase the phosphorylation of several substrates targeted by this complex. At the same time, misplaced PEST phosphatases could target new substrates leading to an aberrant decrease in the phosphorylation of other proteins that normally are not dephosphorylated by PEST phosphatases. Thus, changes in the pattern of protein phosphorylation might explain the contribution of these mutations to different diseases.

In summary, this study provides a detailed description of a new mode of interaction between a Pro-rich peptide and a protein, in this case between the CTH Pro-rich peptide of LYP and the F-BAR domain of PSTPIP1, giving insight into the mechanisms behind autoinflammatory diseases. Furthermore, we identify a group of PSTPIP1 pathogenic mutations related to immune diseases that disrupt this interaction, indicating that the PSTPIP1/phosphatase complex is required for the normal physiology of the immune cells. Finally, the structure of the PSTPIP1/LYP complex will allow predicting the pathogenic effect of any additional PSTPIP1 mutation that could be identified in the future.

### Supplementary Information

Below is the link to the electronic supplementary material.

Supplementary file1 (PDF 1644 KB)

## Data Availability

Coordinates and structure factors have been deposited in the Protein Data Bank (PDB) under the accession codes 7AAL (PSTPIP1-G258A), 7AAN (PSTPIP1), and 7AAM (PSTPIP1/LYP complex); the raw diffraction images have been deposited in the Zenodo repository (https://doi.org/10.5281/zenodo.3874282, https://doi.org/10.5281/zenodo.3876142 and https://doi.org/10.5281/zenodo.3876245). Other data are available from the corresponding author.
