# Supplementary material for: PSTPIP1-LYP phosphatase interaction: structural basis and implications for autoinflammatory disorders
Source: Cell Mol Life Sci. 2022 Feb 12;79(2):131. doi: 10.1007/s00018-022-04173-w (PMC8840930; doi:10.1007/s00018-022-04173-w)
Supplement: Supplementary file 1 — Supplementary file1 (PDF 1644 KB) [file 18_2022_4173_MOESM1_ESM.pdf]

Supplementary Materials for

PSTPIP1-LYP phosphatase interaction: structural basis and implications for autoinflammatory disorders

José A. Manso[2], Tamara Marcos[1], Virginia Ruiz-Martín[1], Javier Casas[1] Pablo Alcón[2], Mariano Sánchez Crespo[1], Yolanda Bayón[1], José M. de Pereda[2] and Andrés Alonso[1*]

[1]Unidad de Excelencia Instituto de Biología y Genética Molecular (IBGM), CSICUniversidad de Valladolid, c/ Sanz y Forés 3, 47003 Valladolid, Spain.

[2] Instituto de Biología Molecular y Celular del Cáncer (IBMCC), CSIC-Universidad de Salamanca, Campus Unamuno, 37007, Salamanca, Spain

∗Corresponding author. Email: andres@ibgm.uva.es

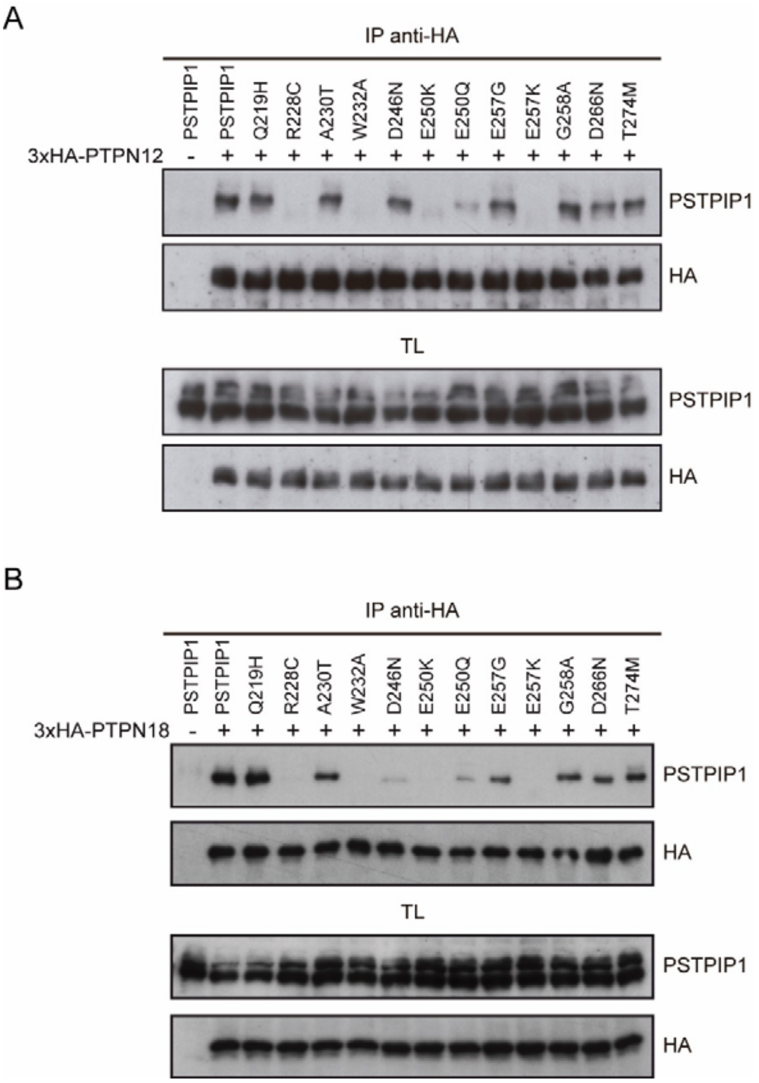

Fig. S1. Interaction of PTP-PEST and PTPN18 with several PSTPIP1 mutants. Analysis of the interaction of PSTPIP1 mutants with PTP-PEST (**A**) and with PTPN18 (**B**), respectively, as indicated previously for LYP in Fig. 1.

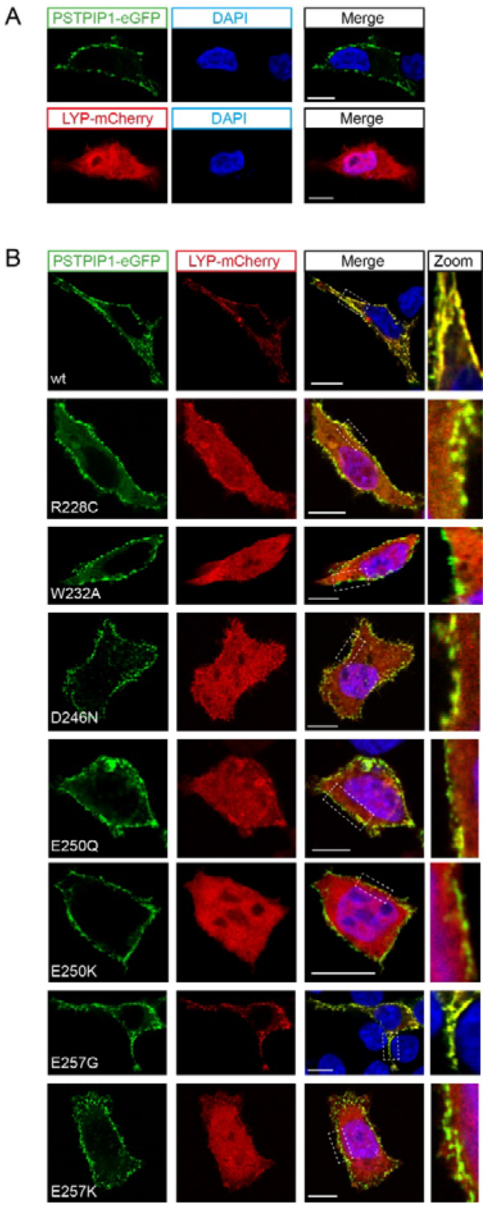

Fig S2. **Subcellular distribution of PSTPIP1 and LYP in HEK 293 cells A)** Representative confocal images of HEK 293 cells expressing wt PSTPIP1-eGFP (top) or LYP-mCherry (bottom) and nuclei counterstained with DAPI are shown. **B)** Representative HEK293 cells co-transfected with LYP-mCherry (red) and wt or indicated mutants of PSTPIP1-eGFP (green) were counterstained with DAPI and analyzed by confocal microscopy. Separate channels, including merge and a membrane magnification of the framed zones in the merge images are shown (right panels). Scale bar represents 10 μm.

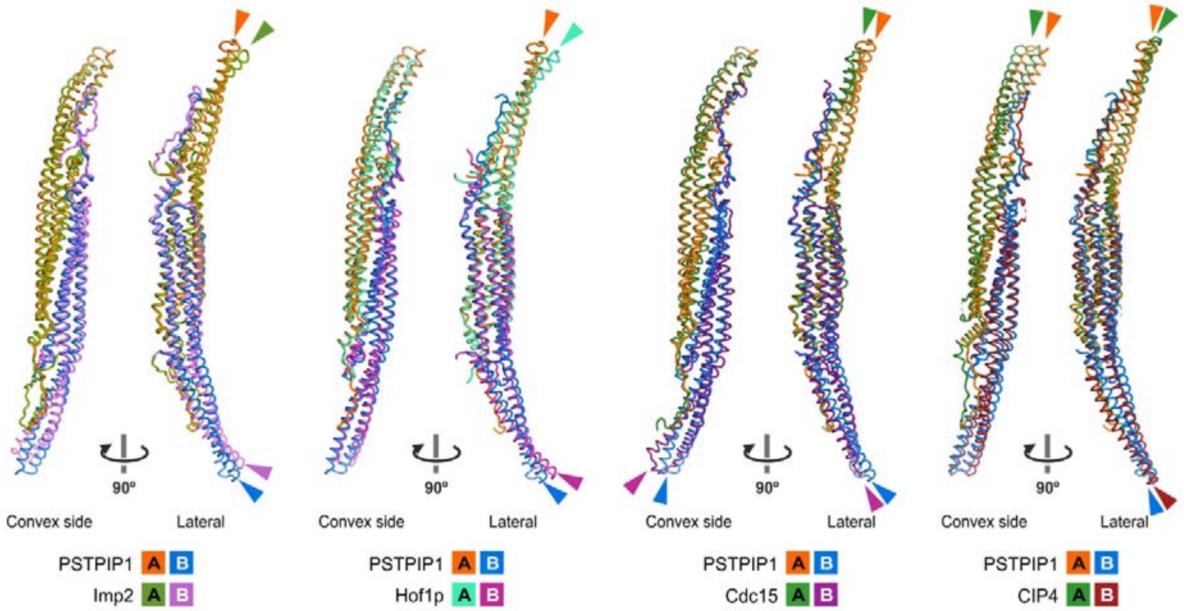

Fig. S3. Comparison of the F-BAR domains of PSTPIP1 and other proteins. Superimposition of the dimers of Imp2, Hof1p, Cdc15 and CIP4 onto the PSTPIP1 structure. The two protomers of each dimer are colored as indicated. Arrows mark the position of the tips of the α3-α4 wings.

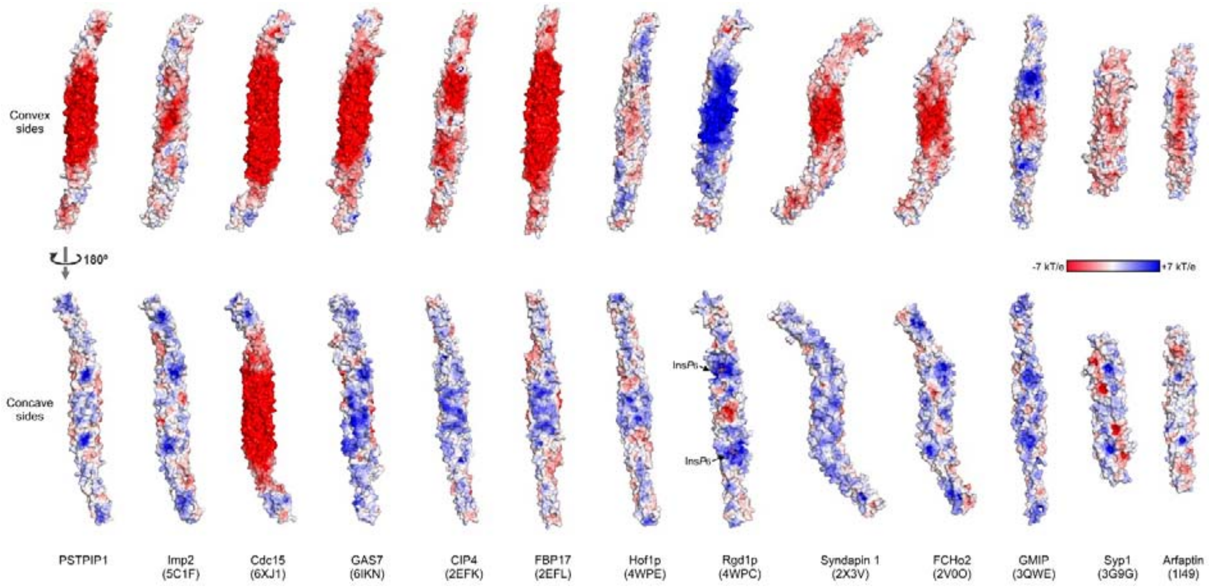

Fig. S4. Comparison of the electrostatic properties of PSTPIP1 and other F-BAR domains. Representation of the molecular surfaces of F-BAR dimers of PSTPIP1 colored by the electrostatic potential mapped on the surfaces. Each structure is shown in two orientations that correspond to the view of the convex (top) and the concave (bottom) sides. The PDB codes of the structures are indicated in parenthesis. The Cdc15 structure was determined for the E30K/E152K mutant and was reverted to the wild type sequence before calculation. The *myo*-inositol-1,2,3,4,5,6-hexakisphosphate (InsP_6) molecules bound to Rgd1p are shown as sticks, but were not included in the calculations of the electrostatic potential.

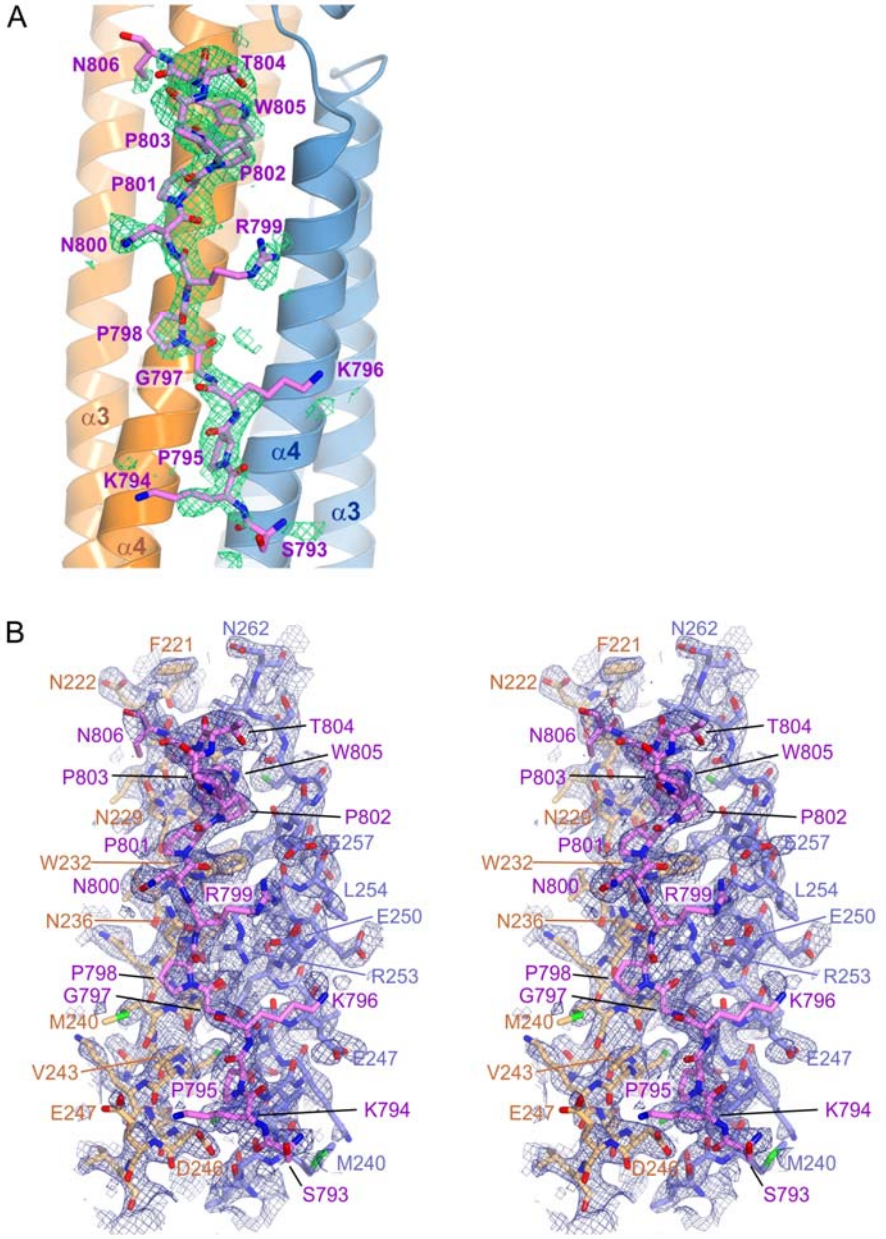

Fig. S5. Electron density maps around LYP in the structure of the PSTPIP1/LYP complex. (**A**) Close up of the structure of LYP (sticks) bound to PSTPIP1 (ribbons). A *mFobs-DFcalc* difference map (green mesh) calculated for a partially refined structure, in which LYP had not been modelled yet, is shown contoured at 2.0 σ around LYP. (**B**) Stereo view of a *2mFobs-DFcalc* composite omit map contoured at 1.0 σ around LYP and PSTPIP1 (shown as sticks). Model bias was reduced by combining areas of multiple maps; each of them calculated after omitting 5% fragments of the structure and refining the incomplete models with a simulated annealing protocol.

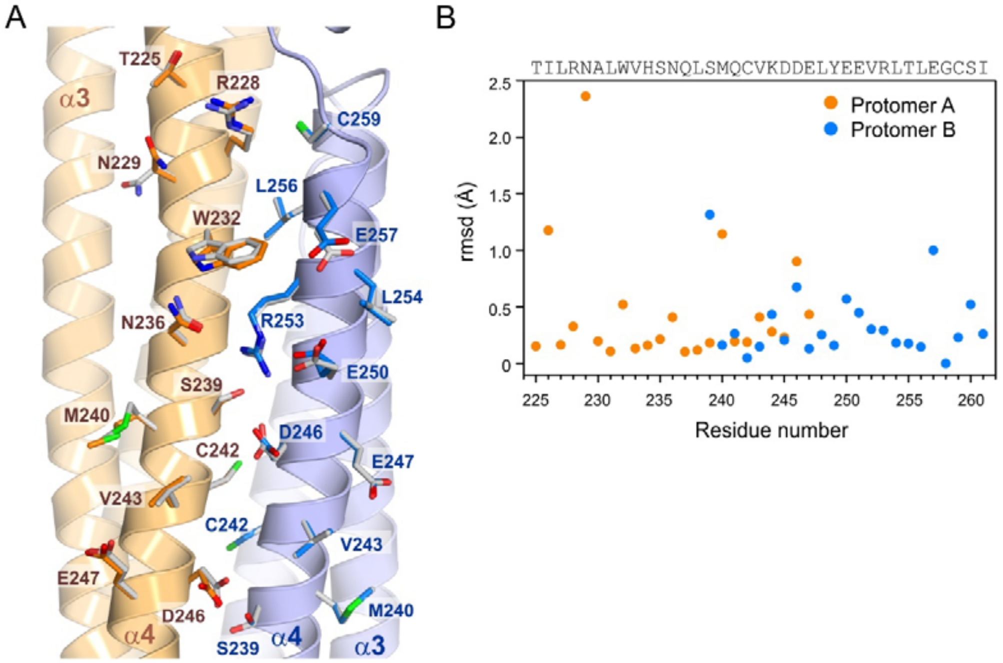

Fig. S6. Comparison of the LYP-binding site of PSTPIP1 in the free and LYP-bound structures. (**A**) Ribbon representation of the structure of PSTPIP1 bound to LYP; the side chains of residues in the LYP-binding interface are shown as sticks. Protomers A and B of the PSTPIP1 dimer are coloured orange and blue, respectively. The side chains of those residues in the unbound structure of PSTPIP1 are shown in grey. (**B**) Average per residue root mean square deviation (rmsd) of the atoms of the side chains, between the free and LYP-bound structures of PSTPIP1.

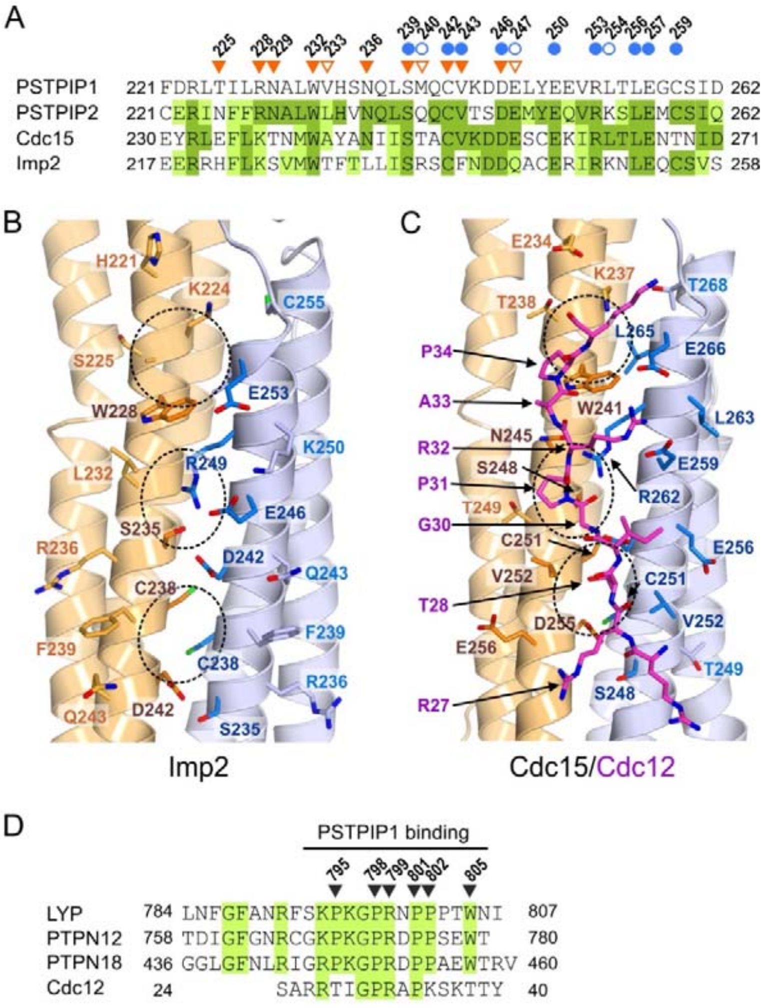

Fig. S7. The LYP-binding site of PSTPIP1 is conserved in Cdc15 and Imp2. (A) Alignment of the region of the sequences of PSTPIP1, PSTPIP2, Cdc15 and Imp2 that correspond to the LYP-binding site in PSTPIP1. Residues from protomers A and B in PSTPIP1, which form the interface, are indicated by inverted orange triangles and blue circles, respectively. Amino acids whose solvent exposed surface was buried ≤ 20% by LYP are indicated by open symbols. Residues identical to those of PSTPIP1 are shown by dark green boxes, while conserved positions are marked by light green boxes. (B) Representation of the region of the structure of the F-BAR domain of yeast Imp2 (PDB ID 5C1F) equivalent to the LYP-binding site of PSTPIP1. The side chain of residues equivalent to those of PSTPIP1 involved in the LYP-binding interface are shown as sticks. Positions identical in Imp2 and PSTPIP1 are shown in darker colors. The three shallow pockets equivalent to those in PSTPIP1 are marked by dashed lines. (C) Model of the Cdc15/Cdc12 complex. Cdc12 was modeled onto the structure of the F-BAR domain of Cdc15 (PDB ID 6XJ1) using the structure of LYP bound to PSTPIP1. Residues and pockets in the predicted binding site are labeled as in B. (D) Multiple sequence alignment of the Cdc15-binding segment of Cdc12 and the CTH domains of PTP-PEST phosphatases. Residues of LYP that make specific contacts with PSTPIP1 are indicated by inverted triangles. Conserved residues are shown in green boxes.

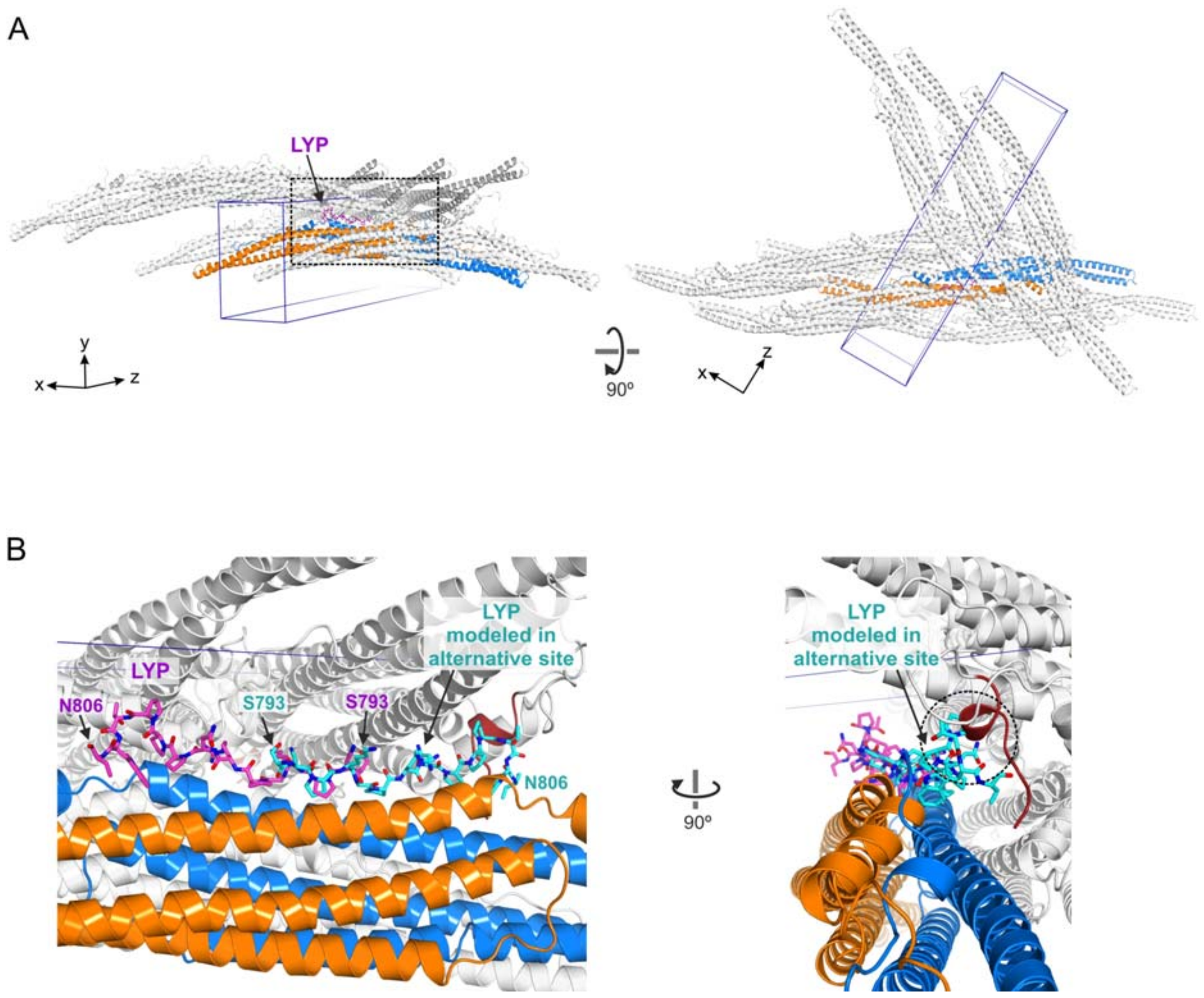

Fig. S8. Crystal packing prevents LYP binding to one of the symmetry related sites in the PSTPIP1 dimer. (A) Two views of the arrangement of the PSTPIP1-LYP complexes in the crystal. The two protomers of the PSTPIP1 dimer and the molecule of LYP that constitute an asymmetric unit of the crystal are colored blue, orange, and magenta, respectively. Symmetry related copies of the complex are colored grey. Blue lines mark the limits of the unit cell. (B) Close up of LYP (magenta) bound to PSTPIP1 (blue and orange) in an asymmetric unit, and neighboring molecules in the crystal (grey). For comparison, a second molecule of LYP (cyan) is shown modeled bound to the alternative binding site of the PSTPIP1 dimer; which was not observed in the crystal structure. LYP bound in this second orientation would clash with the N-terminal helix (dark red) of another PSTPIP1 molecule of the crystal, highlighted by a dashed circle.

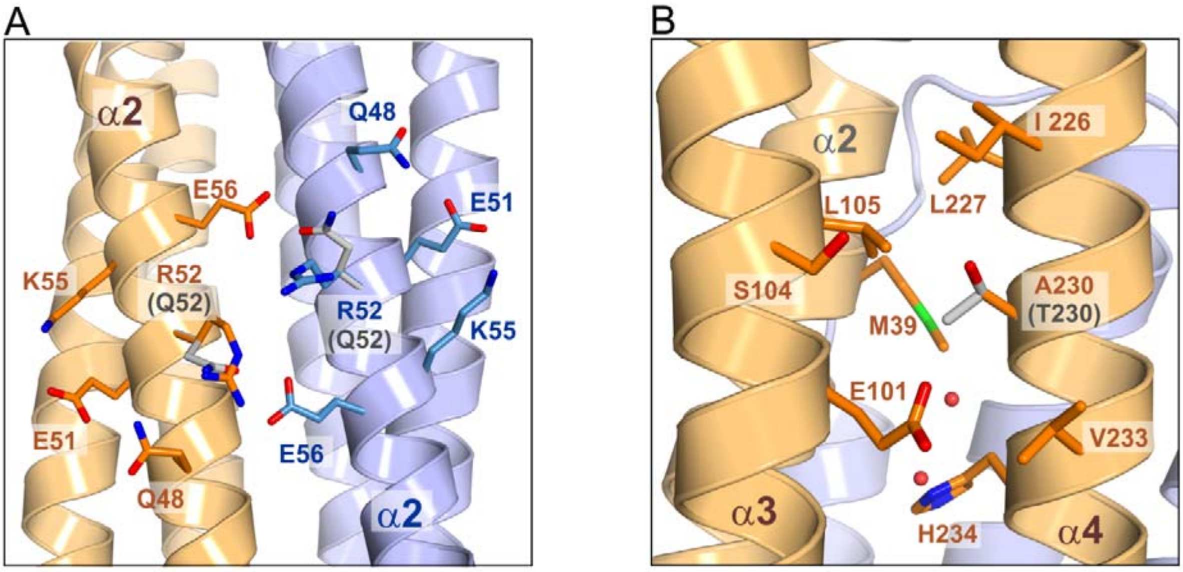

Fig. S9. Structural modeling of PSTPIP1 mutations R52Q and A230T. Close up views of the structure of the F-BAR showing the structural environment of the residues R52 (**A**), and A230 (**B**). The side chains of these and other residues in their surroundings are shown as sticks. Protomers A and B of the F-BAR dimer are shown in orange and blue colors, respectively. The side chains of the residues introduced by mutations R52Q and A230T were modeled as energy-favorable conformations that do not clash with neighboring atoms; they are shown in grey and are labeled in parenthesis.

Table S1.

Table S1. Crystallographic data collection and refinement statistics

	PSTPIP1 F-BAR (1-289) G258A	PSTPIP1 F-BAR (1-289)	PSTPIP1 F-BAR (1-289) / LYP
**Data collection**			
X-ray source	Diamond, I03	Diamond, I03	Diamond, I03
Space group	$P\ 2_1\ 2_1\ 2_1$	$P\ 2_1\ 2_1\ 2_1$	$P\ 2_1\ 2_1\ 2_1$
Unit cell dimensions	a=48.2 Å<br>b=73.0 Å<br>c=205.3 Å	a=48.3 Å<br>b=71.9 Å<br>c=204.6 Å	a=48.0 Å<br>b=72.0 Å<br>c=205.0 Å
Wavelength (Å)	0.99987	0.99987	0.99987
Resolution (Å) [a]	1.97 / 2.92 / 2.09<br>(2.17-1.97) [b]	2.12 / 4.32 / 2.17<br>(2.33-2.14)	2.11 / 4.05 / 2.10<br>(2.32-2.15)
Unique reflections	30894	18546	20328
Average multiplicity	12.3 (9.9)	19.1 (17.7)	19.1 (17.9)
Completeness, spherical (%)	59.4 (12.1)	46.1 (10.4)	51.5 (13.4)
Completeness, ellipsoidal (%)	90.0 (49.4)	91.4 (65.2)	91.9 (61.3)
R_{meas} [c]	0.143 (1.580)	0.428 (3.235)	0.432 (3.172)
CC 1/2	0.998 (0.481)	0.996 (0.540)	0.995 (0.521)
Mean $I/\sigma I$	13.1 (1.6)	7.5 (1.4)	8.0 (1.4)
**Refinement**			
Resolution range (Å)	103 – 1.97	102 – 2.18	103 – 2.15
Unique reflections, work/free	29314 / 1568	17579 / 956	19272 / 1042
R_{work} / R_{free} [d] (%)	18.6 / 21.9	21.1 / 24.1	20.5 / 24.1
Number of<br>residues<br>waters<br>other	 285 / 285<br>310<br>-	 285 / 286<br>79<br>-	 285 / 287 / 14<br>162<br>2
Average B value ($Å^2$)<br>Wilson plot<br>protein<br>waters<br>other	 30.5<br>36.8 / 40.3<br>39.4<br>-	 27.7<br>37.3 / 39.5<br>23.8<br>-	 24.4<br>32.1 / 34.5 / 35.7 25.5<br>47.4
rmsd bond lengths (Å)	0.001	0.001	0.001
rmsd angles (°)	0.37	0.29	0.32
Ramachandran plot [e], residues in<br>Favored regions<br>Additionally allowed<br>Outliers	 574 (99.3%)<br>4 (0.7%)<br>0	 559 (98.6%)<br>8 (1.4%)<br>0	 576 (98.3%)<br>10 (1.7%)<br>0
PDB ID	7AAL	7AAN	7AAM

[a] Resolution limits of ellipsoid fitted to diffraction cut-off surface along reciprocal axis a*, b*, and c*
[b] Numbers in parenthesis correspond to the outer resolution shell.
[c] R_{meas} is the multiplicity independent R factor (68).
[d] Calculated using 5% of reflections that were not included in the refinement.
[e] As defined in the program MOLPROBITY (69).

Table S2. Thermodynamic parameters of the LYP binding to the F-BAR of PSTIPIP1 determined by ITC.

Experiment	C_{LYP} [a] (μM)	$C_{PSTPIP1}$ [a] (μM)	Stoichiometry (N)	K_d (nM)	ΔH (kcal mol^{1})	ΔS (cal mol^{-1} deg^{-1})
1	146.8	28.0	0.50 ± 0.01	263 ± 21	-16.7 ± 0.2	-25.8
2	138.4	26.2	0.49 ± 0.01	229 ± 18	-16.2 ± 0.1	-24.0
3	155.2	30.6	0.46 ± 0.01	267 ± 21	-16.4 ± 0.1	-24.8

[a] The concentrations of PSTPIP1 (residues 1-289) and LYP (residues 787-807) refer to the initial concentrations in the cell and the syringe, respectively.
